# Strategic leadership in elite sport organizations: a systematic review of governance, leadership, and performance systems

**DOI:** 10.3389/fspor.2026.1780254

**Published:** 2026-07-28

**Authors:** Abdelaziz Qarouach, John Cairney, Nadia Saqri

**Affiliations:** 1 Education Sciences Department, Normal Superior School ENS, University Hassan 2, Casablanca, Morocco; 2 Queensland Centre for Olympic and Paralympic Studies, School of Human Movement and Nutrition Sciences, University of Queensland, St Lucia, QL, Australia; 3 Education Sciences Department, Normal Superior School ENS, University Hassan 2, Casablanca, Morocco

**Keywords:** elite sport, governance, performance systems, sport organization, strategic leadership

## Abstract

**Background:**

Strategic leadership describes the various leadership styles and how different countries succeeded in international sports competitions. Leadership success is clearly determined by three inputs: a strategic vision, efficient implementation, and performance impact, i.e., increasing a country's international influence in sport. To categorize the most common research topics, main findings, and shortcomings of analyses conducted in sport organizations, a systematic review was carried out.

**Methods:**

Based on the PRISMA protocol, relevant articles published from January 2010 to August 2025 were searched in Scopus, Web of Science, and PubMed. After screening 309 records, 38 empirical studies were included. The findings indicate that strategic leadership varies across international contexts and has received increased attention since 2010.

**Results:**

The selected studies reveal three interdependent pillars: (1) contingent governance structures, (2) culturally mediated leadership, and (3) configurational performance systems. A reliance on cross-sectional, self-reported data limits causal claims. Future research requires longitudinal designs and objective measures.

**Conclusion:**

The information reported may be of great interest to leaders, contributing to a better characterization of strategic leadership in sport organizations and the subsequent development of training enhancement programs. Additionally, the summary and classification provided may serve as a useful guide for future research in sport leadership.

## 1 Introduction

Organizations worldwide have undergone significant transformations in recent decades, evolving toward more decentralized structures. A notable shift has occurred from a focus on management to an emphasis on leadership (Mazarei et al. (45)). Although managerial and visionary leadership are often treated as distinct continuums, their integration forms the basis of strategic leadership. Strategic leadership is defined as the ability to influence others to make daily decisions that ensure an organization's long-term growth and survival while maintaining short-term financial health (Ru and Duan, (2)). Effective strategic leaders implement diverse strategies to foster a forward-looking perspective, articulate a clear vision, and transform organizational learning practices. The sports ecosystem has experienced rapid changes, demanding adaptive and strategic responses from sports organizations (federations, leagues, clubs, and associations) in an increasingly unpredictable environment. Fluctuations in game conditions, team performance, and organizational factors require sport bodies to adopt complex, globally oriented strategies (1). Consequently, there is a growing need for leaders who can develop and execute innovative strategies (Saber et al. (47)). Sports federations, key entities in governing competitive sports domestically, require strategic leadership that integrates management and leadership approaches to foster long-term growth and address nationwide challenges. Such leadership involves visioning, environmental scanning, collaborative planning, and empowered decision-making, enabling federations to navigate complexity and meet multi-stakeholder needs (2). An unresolved tension in the strategic leadership literature concerns the relationship between operational and visionary dimensions. Ru and Duan (46) emphasize daily decision-making that ensures organizational survival, while Goudarzi and Hamidi (2) foreground vision formation as the core strategic act. Rather than treating these as contradictory, we conceptualize strategic leadership as a dual-process phenomenon: visionary leadership establishes long-term direction and meaning, while operational strategic leadership translates vision into actionable routines and resource allocations (43). In sport organizations, this duality manifests in board-level strategic planning (visionary) and performance management systems (operational), both of which are addressed in our review. Meanwhile, we consider that strategic leadership is not limited to federations as central organizations but also extends to smaller ones, including regional leagues, local clubs, as well as nonprofit community organizations. In short, it applies to all organizations substantially linked to high-performance sport environments. Cultivating strategic leadership in sports can enhance competitive performance, promote inclusion, and support grassroots participation. Existing leadership models, such as those analyzing levels (micro, meso, macro) from De Bosscher et al.'s (3) work, provide a foundational framework. To enable a more accurate reading of the review, we explicitly articulate four theoretical frameworks. First, institutional theory (Scott 2014) explains how normative pressures from governments, sponsors, and international federations shape governance structures. Second, resource dependence theory (Salancik and Pfeffer, 48) illuminates power dynamics between federations, clubs, and leagues regarding resource allocation. Third, contingency theory (4) provides the foundation for our model's proposition that no single governance template fits all contexts. Fourth, configurational theory (5) supports the principle of equifinality: multiple organizational configurations can achieve high performance. However, the effectiveness of strategic leadership is influenced by multiple factors, including organizational size, structure, resources, and external variables such as uncertainty, competition, and sociocultural context (Samimi et al. 49). Organizational variables such as strategic orientation, human resource development, learning strategy, and ethical strategy play critical roles (6). In this review, we adopt an organizational-level conceptualization of strategic leadership, defined as the configuration of governance structures, leadership practices, and strategic systems that collectively enable sport organizations to formulate and implement long-term direction. This perspective extends beyond individual leader traits to encompass distributed leadership, board-level decision-making, and institutional mechanisms (7, 43). Despite this, recent studies identify a significant gap in sports federations: there is still a lack of strategic leadership thinking. This deficiency hinders the ability of organizations to thrive in dynamic environments and achieve long-term goals (Parent et al. (50)). To theorize how organizations, as distinct from individuals, exercise strategic leadership, we draw on three complementary perspectives. First, following Ferkins et al. (7), governance structures themselves embody strategic leadership through board composition, decision-rights allocation, and accountability mechanisms. Second, drawing on De Bosscher et al.'s (3) SPLISS model, strategic leadership operates across micro (individual leader), meso (organizational), and macro (national policy) levels. Within this framework, organizational-level strategic leadership is operationalized through resource configuration, policy implementation, and coordination mechanisms across federations, clubs, and leagues. Third, institutional theory suggests that organizations “lead” by shaping the normative and cognitive frameworks within which individual leaders act (Scott 2014). That said, strategic thinking is not only essential for adapting to change but also for driving sustainable evolution within national sports systems. For this reason, this literature review aims to explore the impact of strategic leadership across different countries and sport types. The central research question is: *how can strategic leadership ensure sport development and promotion?* The review addresses the following research questions:

1. What organizational conditions (governance structures, leadership development systems, strategic performance mechanisms) shape strategic leadership practices in elite sport organizations (federations, clubs, leagues)?
2. What are the key findings, prevalent research topics, and methodological limitations of empirical studies investigating the relationship between organizational conditions and performance outcomes in elite sport?
3. What theoretical and empirical gaps exist in the field of organizational strategic leadership in elite sport, and what integrated model can synthesize current evidence?

By systematically reviewing and organizing the existing literature, this study seeks to identify common themes, synthesize findings, and highlight areas for future research. Understanding the evidence base for strategic leadership in sport, including sample characteristics, research aims, and variables studied, will assist in optimizing future research designs and support leaders in refining leadership development processes.

## 2 Methods

### 2.1 Review aim and design

This systematic review aims to synthesize the extant literature on strategic leadership within sport organizations. Its primary objective is to examine the effectiveness of strategic leadership in relation to sport participation, organizational management, and overall development. By integrating findings from diverse methodological approaches, this review seeks to provide a comprehensive analysis of strategic leadership's impact and highlight effective strategies across various implementation contexts.

### 2.2 Data sources

To ensure optimal coverage of the existing relevant literature, this review draws on several academic databases. In total, 309 studies were identified in the original database search (Scopus =114; Web of Science = 112; PubMed = 83) to collect local, national, and international studies and research specific to the field of sport leadership. Due to significant heterogeneity in study designs, measured outcomes, and contextual variables across the included literature, a quantitative meta-analysis was not feasible. A narrative synthesis was therefore conducted to identify, analyze, and integrate overarching themes and theoretical propositions, which is an established approach for synthesizing complex, multi-method evidence (Popay et al. 51). While our search strategy included Scopus, Web of Science, and PubMed, we acknowledge the absence of SPORTDiscus, ERIC, and PsycINFO as a limitation. SPORTDiscus is particularly relevant given its sport-specific indexing. Future reviews should prioritize multi-database searches, including SPORTDiscus, to ensure comprehensive coverage. A thematic synthesis (Thomas and Harden, 52) was conducted following three stages: line-by-line coding of findings from all 38 studies; organization of codes into descriptive themes; and development of analytical themes (the three pillars) that go beyond the original studies' findings. Quantitative meta-analysis was not feasible due to: (a) substantial heterogeneity in outcome measures (23 different performance indicators across studies); (b) variability in study designs (cross-sectional, longitudinal, case study, QCA); (c) absence of standardized effect size reporting in 31 of the 38 studies; and (d) conceptual heterogeneity in strategic leadership operationalization. Thematic synthesis is an established approach for integrating multi-method evidence under such conditions (51, 52).

### 2.3 Search strategy

Systematic review principles were employed (8) to conduct a search of three electronic databases (Web of Science, Scopus, and PubMed) using the following keyword combinations: (“sport” OR “athlete” OR “sport policy” OR “sport management” OR “sport governance” OR “sport economy” OR “sport development” OR “sport leadership” OR “sport coaching” OR “elite sport” OR “youth sport” OR “sport performance” OR “high performance” OR “sport participation” OR “Sport education”) AND (“strategic governance” OR “strategic management” OR “strategic policy” OR “strategic leadership” OR “talent” OR “infrastructure” OR “funding” OR “gender” OR “women” OR “disability” OR “rural” OR “community sport” OR “public sport” OR “sport system” OR “sport federation” OR “sport organization” OR “sport club”).

### 2.4 Inclusion and exclusion criteria

To specifically target studies dealing with strategic leadership in sport, the searches were restricted to articles that met the following inclusion and exclusion criteria:

Inclusion: For the purpose of this review, “elite sport” is operationally defined as: (a) sport contexts involving national or international competition at the highest level (e.g., Olympic Games, world championships, professional leagues); (b) organizations responsible for high-performance athlete development (e.g., national governing bodies, professional clubs, Olympic federations); and (c) systems explicitly designed to achieve podium-level outcomes. Studies focused exclusively on recreational, grassroots, or developmental sport without explicit elite pathways were excluded unless they directly addressed strategic leadership mechanisms directly applicable to elite contexts (Table 2).

Exclusion: (a) Nonempirical studies (e.g., pure commentary); (b) studies in contexts not involving sport organizations (e.g., corporate); (c) studies that were not peer-reviewed (grey literature); and (d) studies for which the full text was unavailable.

The research was conducted from 1 January 2010 to 14 August 2025. The rationale for the period (2010–2025) was based on two theoretical justifications: the publication of De Bosscher et al.'s (9) foundational SPLISS framework marked a turning point in systematic elite sport policy research; and a pilot scoping search revealed that fewer than 8% of relevant studies on strategic leadership in sport organizations were published before 2010.

### 2.5 Data extraction strategy

Data extraction was standardized and systematic to ensure consistency and rigor in the collection of information relevant to this systematic review. Extracted data included information on the full study reference, the type of study (implementation, development, or evaluation, etc.), the study population (local, national, and international), as well as a detailed description of the adopted leadership approach used. In addition, measured outcomes (participation, improvement, and performance) were extracted and analyzed. This extraction enabled in-depth analysis of the data and ensured transparency in the collection process. The results were then synthesized according to their methodological quality and their impact on the research question.

### 2.6 Quality appraisal

The methodological quality of the 38 included studies was appraised using the Mixed Methods Appraisal Tool (MMAT), version 2018. The MMAT allows for the concurrent appraisal of qualitative, quantitative, and mixed-methods studies. Two reviewers independently assessed each study. Inter-rater agreement was calculated using Cohen's kappa (*κ* = 0.88). Disagreements were resolved through discussion or by consultation with a third reviewer. No studies were excluded based on quality scores; instead, the appraisal informed the critical synthesis of findings and the weighting of conclusions.

## 3 Results

### 3.1 Identification and selection of studies

**Figure 1:**
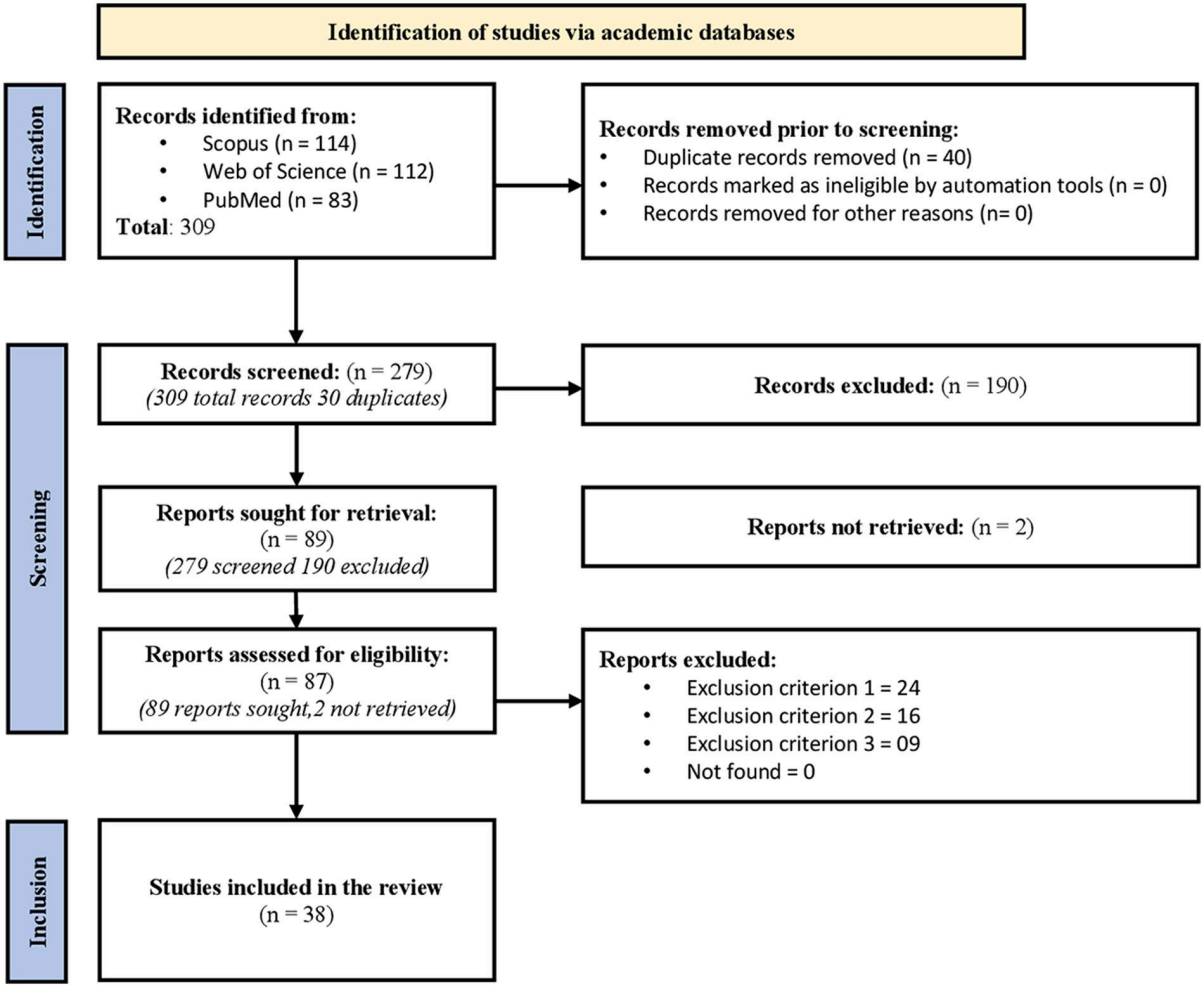
Systematic review flowchart.

We used PRISMA (Preferred Reporting Items for Systematic Reviews and Meta-Analyses) flow diagram to summarize the search results. In total, 309 studies were identified in the original database search (Scopus = 114; Web of Science = 112; PubMed=83). After removing duplicates using a computer-based reference management system (EndNote X6, Thomson Reuters, New York, USA), two individual researchers performed the first stage screening of titles and abstracts against the eligibility criteria for 279 studies. Authors of the publications were masked from the reviewers. References not eliminated were subjected to second-stage screening of the full text based on the inclusion and exclusion criteria. To ensure the quality of the review process (8) a measure of agreement between the two individual researchers was performed using Cohen's kappa calculation. Scores of *κ* = 0.91 and *κ* = 1.00 were recorded for the first- and second-stage screening, respectively. Disagreements were resolved by discussion or via a third researcher. Finally, to ensure a relatively complete census of the relevant literature, one researcher performed a backward and forward reference search*,* reviewing the references and citations of the included studies (10). Moreover, a second-level backward reference search was conducted by pulling the references of the references (11). At the end of the process, a total of 38 studies were included in the current systematic review. The results of this section are structured around three main areas of analysis (Figure 1). First, the identification of studies via academic databases (Scopus, Web of Science, and PubMed) is highlighted, along with the process of searching for and selecting relevant work from the 309 studies identified in the three databases. Second, in the screening phase, the sport implementation contexts of the studies are examined, with sample profiles, such as contexts of implementation, objectives, and main purposes, being analyzed (89 selected studies). Finally, a synthesis of the studies with higher inclusive criteria is provided, analyzing their objectives, the methodologies employed, and the main results obtained, thus offering an overview of the scientific and significant contributions (38 studies).

### 3.2 Study characteristics and synthesis

In our results, we provided a general summary of study characteristics, including authors, titles, year of publication, country and level of analysis, theoretical framework, methodology, research objectives, practical implications, and contribution to sport leadership (Tables 1, 2). Additionally, the selected studies are categorized from an organizational standpoint (federation: *n* = 21; professional club: *n* = 12; league: *n* = 5) and by level of competition (Olympic/international: *n* = 24; national elite: *n* = 14), strategic leadership focus, management focus, and Mixed Methods Appraisal Tool (MMAT score, version 2018) (Table 3). We acknowledge that restricting the review to English, French, Portuguese, Greek, and Spanish excludes major bodies of literature in German, Russian, Japanese, and Chinese. This constitutes a limitation, particularly given the prominence of German Olympic sport research and Chinese high-performance systems. However, this restriction was necessitated by the absence of translation resources and the review team's language competencies. Future systematic reviews should incorporate non-English databases to capture region-specific strategic leadership research.

**Table 1:** Summary of included studies 1.

Author(s)	Year	Title	Country context	Level of analysis
Ferkins et al. (7)	2010	A model for improving board performance: the case of a national sport organisations	New Zealand	Organizational (board/NSO) National (New Zealand)
Fletcher and Arnold (30)	2011	A qualitative study of performance leadership and management in elite sport	International	Organizational National
Robinson and Minikin (54)	2011	Developing strategic capacity in Olympic sport organisations	Oceania/Pacific region	Organizations National Federation (NF/NOC)
Arnold et al. (22)	2012	Performance leadership and management in elite sport: recommendations, advice and suggestions from national performance directors	UK	Individual (NPD) Organizational (NGBs) National (Olympic program)
Truyens et al. (55)	2014	A resource-based perspective on countries’ competitive advantage in elite athletics	Belgium	National/sport system (country/sport system level; sport specific: athletics)
Čingienė and Raipa (13)	2015	Implementation of good governance principles: a case of Lithuanian strategic sport federations	Lithuania	Organizational (federation level); Sectoral National context
Naidoo et al. (23)	2015	Perceived leadership styles of sport administrators and the relationship with organisational effectiveness	South Africa	Individual (sport administrators; coaches as raters) Organizational (HEI sport department)
Truyens et al. (21)	2016	An analysis of countries’ organizational resources, capacities and resource configurations in athletics	Belgium, Canada, Finland, Netherlands	National/sport system (country/sport system level; sport specific: athletics)
Frawley et al. (27)	2018	Experience-based leadership development and professional sport organizations	Australia	Organizational
O'Boyle et al. (53)	2018	Identifying enablers and barriers: shaping collaborative sport governance theory	Australia	Organizational (NSO and SSO boards) Sectoral/network (federal sport network)
Clausen et al. (15)	2018	International sport federations’ commercialisation: a qualitative comparative analysis	International	Organizational International (IF level, cross-national comparison of Olympic IFs)
Kasale et al. (17)	2018	Performance management of national sports organisations: a holistic theoretical model	International	Organizational (NSO) with multilevel perspective (macro/meso/micro)
Čingienė and Raipa (13)	2019	Formation of sports public policy within the context of hierarchy governance	Lithuania	National
Hall et al. (20)	2019	The talent development environment questionnaire as a tool to drive excellence in elite sport environments	Hong Kong	Individual Organizational
Mitrovic et al. (26)	2019	The relationship between leadership styles and organisational culture in sport organisations	Montenegro	Individual (members’ perceptions) Organizational (club)
Patrycja (56)	2020	Application of the balanced scorecard in Polish Sports Associations	Poland	Organizational; Sectoral National context (PSAs within Polish national sport system)
Thornton and Etxebarria (28)	2021	Against the odds of tradition: nudging the glass ceiling of sport management and leadership	South-east Asian	Individual Organizational
Svensson et al. (25)	2021	The influence of servant leadership on shared leadership development in sport for development	USA	Organizational National
Djuric et al. (31)	2024	Leadership as critical success factor of total quality and human resource management in sports organizations	Multi countries	Organizational National (Olympic sport programs across national sport systems)
Rodríguez et al. (34)	2022	Evolution and development of the Spanish badminton 2000–2019	Spain	Organizational National (single national federation and system level indicators across Spain)
Glibo et al. (36)	2022	Strategic sustainable development in international sport organisations: a Delphi study	International	Organization International sport organizations
Johnson et al. (44)	2022	Identifying strategic leadership behaviors of sport industry leaders: a phenomenological method	USA	National
Şahin (60)	2022	Effective leadership types in change management in sports organisations	Turkey	Individual (employees/leader behaviors) Organizational (federation)
Tadesse et al. (24)	2023	Strategic plan and performance of selected Ethiopian sports federations	Ethiopia	Organizational, National (sports federations)
Hatungimana and Oladipo (57)	2023	Talent management processes as predictors of long-term athlete development in sports organisations in Burundi	Burundi	Individual Organizational
Mthombeni et al. (19)	2024	Factors enabling and hindering sporting success among South African elite athletes from historically disadvantaged areas: through a coaching lens	South Africa	National (coach perceptions about national/international athlete success stemming from HDA)
Djuric et al. (31)	2024	Leadership as critical success factor of total quality and human resource management in sports organisations	Serbia	Individual (senior manager respondent) Organizational (club level)
Valentina et al. (58)	2024	Navigating the path to management: insights into sport manager selection and diversity strategies in Romania	Romania	Individual Organizational, National
Morrison and Misener (59)	2024	Strategy practitioners and the procedural legitimacy of strategic planning in nonprofit community sport	Canada	Organizational (community sport organizations/club level) Sectoral (community sport field)
Garmamo et al. (16)	2025	The relationship between strategic management, organisational structure, and good governance in sport in selected Ethiopian Olympic Sports Federations	Ethiopia	Organizational
Tadesse et al. (24)	2024	Transformational leadership on performance of selected Ethiopian sport federations	Ethiopia	Individual (employees’ perceptions) Organizational (performance of selected sport federations)
Kohandel et al. (6)	2024	Developing a strategic leadership model for the Iranian sports federations	Iran	Organizational National (strategic leadership in national sports federations)
Cairney et al. (29)	2024	Toward a knowledge synthesis heuristic for sport leaders: the strategic leader synthesis model	Australia	Conceptual Perspective
Radišić et al. (32)	2024	Success of sports organizations and correlation with efficiency and leadership style	Serbia	Individual (managers/respondents) Organizational (club, Organization)
Todaro et al. (37)	2025	Enablers and constraints of environmental sustainability integration: structuration perspective on professional sports organisations	European	Organizational Sectoral (NFAs within the European football field)
Ghodhbani and Souissi (14)	2025	Governance of Tunisian sports organisations: what is the matter?	Tunisia	Organizational National (national sports federations; federations as units)
Woldeyes et al. (33)	2025	The impact of strategic management on the competitive performance of Ethiopian Premier League Soccer Clubs	Ethiopia	Organizational (club level; with individual respondents)
Merten et al. (38)	2025	Unlocking value: exploring digital transformation's influence on knowledge management of national football associations	International	Individual (staff) and sectoral implications Organization-level focus on NFAs with cross-national International

**Table 2:** Summary of included studies 2.

Author(s)	Theoretical framework	Methodology	Research objective/aim	Practical implications	Contribution to sport leadership
Ferkins et al. (7)	Action research; institutional theory; stakeholder theory; governance (performance vs. conformance)	Qualitative	To develop and test a structured four-stage action research model	Managerial actions validated: implement district mentoring and capacity building	Provides an applied action research model for improving strategic capability and sport governance
Fletcher and Arnold (30)	Transformational leadership; leader effectiveness; functional leadership	Qualitative	To identify perceptions of performance leadership and management in elite sport	Aligns leadership findings with existing leadership theories	Test differentiated models of leadership in elite sport
Robinson and Minikin (54)	Institutional theory; organizational development	Mixed	To identify performance dimensions in NFs; hierarchical development levels	Produced a practical diagnostic matrix	Introduces a practitioner-oriented, empirically grounded Assessment Tool
Arnold et al. (22)	Organizational model; situational leadership; performance management stakeholder	Qualitative	To elicit stakeholder driven recommendations for performance leadership and management in elite sport	Practical checklist of five focused areas	Provides empirically grounded, stakeholder-derived taxonomy of leadership and organizational elite sport
Truyens et al. (21)	Elite sport policy and management	Qualitative	To identify organizational resources for competitive advantage in elite athletics	RBV applied at sport-specific (athletics)	Provides the first sport-specific inventory of organizational resources for elite athletics
Čingienė and Raipa (13)	Systems theory/systemic process approach; phenomenological approach	Mixed	To identify and classify key good public governance principles	Strengthen monitoring/audit procedures, communication processes and strategic planning	Provides a focused empirical assessment of good governance principles in national sport federations
Naidoo et al. (23)	Leadership and organizational effectiveness	Quantitative	To assess the relationship between perceived leadership and Organizational effectiveness	Provides empirical backing in HEI sport leadership context	Mediating the leadership–effectiveness link; cross-cultural comparisons within Africa
Truyens et al. (21)	Mixed: ORFOC framework (derived from SPLISS)	Mixed	To analyze country comparisons of organizational capacity in elite athletics	Confirms RBV/ORFOC claim that resources alone do not guarantee advantage	Apply ORFOC composite indicators longitudinally and to more countries and sports
Frawley et al. (27)	Experience-based leadership development	Qualitative	To investigate the state and nature of leadership development	Create individual leadership plans; senior executives (CEOs)	Provides empirical multi-case evidence of experience-based leadership development
O'Boyle et al. (53)	Collaborative governance	Qualitative	To identify and explore enablers and barriers in federal sport governance networks	NSO, SSO managers' missions	Advances collaborative sport governance
Clausen et al. (15)	Resource dependence theory; institutional theory; stakeholder theory	Mixed	To identify conditions and configurations toward high commercialization	Managerial priorities	Advancing understanding of businesslike and the governance risks
Kasale et al. (17)	Stakeholder theory; Institutional theory	Qualitative	To conceptualize PM for NSOs from a holistic perspective	Provides a practical heuristic (holistic PM model) for sport managers	Propose a holistic, multilevel theoretical PM model
Čingienė and Raipa (13)	Hierarchy governance (authoritarian/hierarchical mode); institutional theory; stakeholder theory	Qualitative	To analyze how Lithuanian sports policymaking peculiarities manifest within hierarchy governance	Managerial implications: implementation and institutional and personal responsibility	Provides a mapped, theory-informed retrospective sports public policy
Hall et al. (20)	Talent Development Environment framework (TDE/Talent environment Theory)	Mixed	To evaluate the use of the TDEQ	The TDEQ can be operationalized as a practitioner tool	Provides a demonstrable applied model
Mitrovic et al. (26)	Perceptions of leadership style and organizational culture	Quantitative	To determine dominant leadership style and the corresponding organizational culture	Empirical support for interactive reciprocity between leadership and organizational culture	Leadership network and the leadership–culture for sport leadership and organizations
Patrycja (56)	Strategic management, stakeholder Theory; institutional theory	Qualitative	To assess adaptation of the Balanced Scorecard (BSC) for Polish Sports Associations	BSC is theoretically suitable for NGOs in sport	Offers concrete indicator recommendations to improve strategic management
Thornton and Etxebarria (28)	Transformational leadership; values-based leadership; institutional theory	Mixed	To capture the participants' leadership journey of female directors in sporting organizations	Supports the importance of values based/transformational leadership in enabling women	Cohort studies evaluating leadership programs; examine interaction of gender with governance quality
Svensson et al. (25)	Servant leadership; shared leadership; vertical leadership	Quantitative	To test servant leadership influences on shared leadership development	Encourage leaders to adopt servant leadership behaviors	Provides first empirical evidence in SFD linking servant leadership to shared leadership
Djuric et al. (31)	Critical realism; systems theory perspective on performance management	Qualitative	To identify which performance management processes are used in Olympic sport programs	Supports systems theory view: performance management	Identifies and organizes performance management processes used in Olympic sport programs
Rodríguez et al. (34)	Harmonized: performance Management, institutional theory	Mixed	To analyze evolution and development of badminton in Spain	Managers and federations priorities	Provides an empirically grounded, multidimensional organizational performance model for a national federation
Glibo et al. (36)	Strategic Sustainable Development (FSSD)	Qualitative	To identify near future strategic priorities for international sport organizations	Managers and federations priorities	Provides a consensus-based set of strategic priorities for development in international sport organizations
Şahin (60)	Trait theory; behavioral theory; contingency/situational leadership; democratic vs. autocratic leadership	Qualitative	To identify types of leadership perceived as effective in sports federations	Supports contingency/situational leadership Theory in sport change contexts	Report the employees in sports federations' view on effective change leadership
Tadesse et al. (24)	Strategic plan	Mixed	To determine the relationship between strategic planning and performance of sports federations	Managers should adopt and invest in strategic planning practices	Provides mixed-method evidence on strategic planning practices with performance in national sports federations
Hatungimana and Oladipo (57)	Talent management	Quantitative	To determine the extent of strategic leadership and long-term athlete development in sports federations	Provide development training for existing personnel	Positive associations between talent management processes and long-term athlete development
Mthombeni et al. (19)	SPLISS nine-pillar framework (De Bosscher et al.)	Qualitative	To evaluate national coaches’ perceptions and experiences of international sporting success by elite athletes	Managers/federations: prioritize subsidized coach education (especially for HDA coaches)	Provides a focused, coach-centered qualitative application of the SPLISS framework
Djuric et al. (31)	Burke Litwin causal model; complexity leadership theory; human and social capital management	Quantitative	To determine which TQM and HRM elements are implemented and test hypothesized	Managerial priorities	Demonstrates higher organizational performance in sports clubs; identifies leadership, mission/vision clarity
Valentina et al. (58)	Multilevel perspective on diversity; Implicit bias theory; boundary policing; institutional dynamics	Qualitative	To investigate sport manager selection and diversity policies in sports organizations,	Recommendations for leadership development	Provides empirical evidence of gap between diversity policy rhetoric and practice in sport management
Morrison and Misener (59)	Institutional theory	Qualitative	To understand the roles and activities of strategy planning	Strategic leadership and alignment with governing body	Provides a grained, practice-oriented account of strategic planning in community sport
Garmamo et al. (18)	Institutional theory; stakeholder theory	Quantitative	To examine strategic management influence in sport governance	Managers should align strategy and structure; invest in strategic management processes	Provides empirical SEM evidence that strategic management positively affects good governance
Tadesse et al. (24)	Transformational leadership	Quantitative	To assess the impact of transformational leadership practices on organizational performance	Behaviors and federation performance and implementing leadership development	Provides evidence within sport federations of transformational leadership with organizational performance
Kohandel et al. (6)	Leadership (strategic leadership); organizational learning; institutional theory	Mixed	To develop and validate a Strategic Leadership model for sports federations	Integrates strategic leadership, organizational learning and absorptive capability ideas	Produces a seven-factor Strategic Leadership model for sports federations
Cairney et al. (29)	Bolman and Deal's Reframing Organizations; Ancona et al.'s 4CAP; network theory	Qualitative	To propose and illustrate a Strategic Leader Synthesis Model for sport leaders	Sport leadership beyond single style and links with capabilities and mindset literatures	Validate and refine model for capabilities and mindsets; links to strategic leadership
Radišić et al. (32)	Transformational leadership; transactional leadership; Laissez-faire; organizational effectiveness	Quantitative	To determine the structure and management leadership styles on success of sports organizations	Empirical support for situational/structural management and organizational success	Provides an empirical analysis of management characteristics in perceived organizational success
Todaro et al. (37)	Structuration theory, institutional theory and stakeholder perspectives	Qualitative	To identify factors in transforming signification, legitimation, and domination structures	Managerial: Top management endorsement is critical across modalities	Operationalizes Giddens’ modalities for ES integration in sport
Ghodhbani and Souissi (14)	Stakeholder theory; Bayle's governance typology; institutional theory	Quantitative	To identify and categorize governance modes	Adoption of governance models that align with operational needs and strategic goals	Provides a systematic empirical test linking Bayle's governance typology to organizational performance
Woldeyes et al. (33)	Strategic management	Quantitative	To examine strategic management practices (governance)	Clubs should prioritize formal strategic planning	Provides an empirical quantification of comprehensive strategic management
Merten et al. (38)	Knowledge-based view (KBV)	Qualitative	To assess the impact of DT on KM elements and effect NFAs’ value creation	NFA managers should invest in digital infrastructure and staff training	Conceptualizes digital strategic use and identifies human resources and governance

**Table 3:** Categorized studies according to level of competition, strategic leadership focus, and management focus.

Number	Author	Country	Organization type	Level of competition	Strategic leadership focus	Management focus	MMAT score
1	Arnold et al. (22)	UK	National Federations	Olympic	Vision, culture, people, relational skills	Operations, functional management	4/5
2	Cairney et al. (29)	Canada/Australia	Multi-sport organizations	International	Strategic leader synthesis model	Knowledge synthesis heuristic	4/5
3	Cingienė and Raipa (13)	Lithuania	Sport Federations	National	Governance transparency, democratization	Bureaucratic control, procedural compliance	3/5
4	Clausen et al. (15)	Multiple (Europe)	International Federations	Olympic	Strategic commercialization paths (QCA)	Resource allocation, staff specialization	5/5
5	De Bosscher et al. (3)	Multiple	National Sport Systems	Olympic	Macro-level strategic leadership (SPLISS)	Policy implementation, resource configuration	5/5
6	Djuric et al. (31)	Serbia	Tennis Clubs	National	TQM/TQHRM leadership as critical success factor	Total quality management, human resource systems	4/5
7	Ferkins et al. (7)	New Zealand	National Sport Organization	National	Board strategic leadership, shared understanding	Governance processes, board composition	4/5
8	Fletcher and Arnold (30)	UK	Olympic Programs	Olympic	Performance leadership domains	Management systems integration	4/5
9	Frawley et al. (27)	Australia	Professional Sport Organizations	Professional	Experiential leadership development	Cross-functional projects, crucible experiences	3/5
10	Garmamo et al. (16)	Ethiopia	Olympic Federations	Olympic	Strategic management, governance fit	Structural alignment, good governance practices	4/5
11	Garmamo et al. (18)	Ethiopia	Olympic Federations	Olympic	Mediation role of organizational structure	Strategic planning, implementation, control	4/5
12	Ghodhbani and Souissi (14)	Tunisia	Sport Organizations	National	Contingency presidential models (strong, managerial, couple-based)	Governance architectures	4/5
13	Glibo et al. (36)	Multiple	International Sport Organizations	Olympic	Strategic sustainable development priorities	Bidding processes, event management	4/5
14	Hall et al. (20)	UK	Elite Sport Environments	Olympic	Talent Development Environment (TDEQ) as strategic tool	Ecological measurement, intervention design	5/5
15	Kasale et al. (17)	Botswana	National Sport Organizations	National	Holistic performance management model (macro/meso/micro)	Feedback loops, performance systems	4/5
16	Kohandel et al. (6)	Iran	Sport Federations	National	Strategic leadership model (vision, control, HR, ethics)	Operational planning, resource development	4/5
17	Merten et al. (38)	Multiple	National Football Association	Professional	Digital knowledge management as strategic lever	Knowledge creation, storage, transfer, application	4/5
18	Mitrovic et al. (26)	Montenegro	Sport Clubs	National	Team management style (task+people orientation)	Clan culture, trust, loyalty, member development	3/5
19	Molan et al. (35)	Multiple	Olympic Sport Programs	Olympic	Dynamic system of interrelated processes	Strategic, operational, individual level linkages	4/5
20	Mthombeni et al. (19)	South Africa	Elite Athlete Development	International	Factors enabling/hindering success (coaching lens)	Talent identification, coach provision, scientific support	3/5
21	Naidoo et al. (23)	South Africa	Sport Administrators	National	Transformational leadership dominance	Organizational effectiveness, management systems	3/5
22	O’Boyle et al. (53)	Australia	Federal Sport Networks	National	Collaborative governance regimes	Board composition, voting systems, leadership style	4/5
23	Parent et al. (50)	Canada	Sport Federations	National	Strategic leadership thinking (gap analysis)	Long-term goal alignment	4/5
24	Radišić et al. (32)	Serbia	Sport Organizations	National	Organizational structure as a stronger predictor than leadership style	Number of employees, role clarity	3/5
25	Rodríguez et al. (34)	Spain	Badminton System	National	SPLISS-informed performance evolution	Resource configuration, competition access	4/5
26	Şahin (60)	Turkey	Sport Organizations	National	Hybrid styles (flexibility, understanding, authority)	Change management, situational models	3/5
27	Samimi et al. (49)	Multiple	Various	Various	Contextual moderators of strategic leadership	Organizational size, structure, resources, uncertainty	5/5
28	Svensson et al. (25)	USA	Sport for Development	Nonprofit	Servant leadership fostering shared leadership	Empowerment, distributed capability	4/5
29	Tadesse et al. (24)	Ethiopia	Sport Federations	National	Transformational leadership and organizational effectiveness	Strategic planning, performance mediation	4/5
30	Thornton and Etxebarria (28)	Multiple	Sport Leadership	International	Women leaders’ career trajectories	International development programs	3/5
31	Todaro et al. (37)	Multiple	Professional Sport Organizations	Professional	Environmental sustainability integration (structuration)	Resource allocation, institutional logics	4/5
32	Truyens et al. (21)	Multiple	Athletics Systems	Olympic	Configurational resource-capability analysis	Talent ID, coach provision, competition access, scientific support	5/5
33	Woldeyes et al. (33)	Ethiopia	Premier League Soccer Clubs	Professional	Strategic management impact on competitive performance	Planning, analysis, implementation, control	4/5
34	Goudarzi and Hamidi (2)	Iran	Sport Federations	National	Vision formation, collaborative planning, empowered decision-making	Environmental scanning, multi-stakeholder needs	3/5
35	Ru and Duan (46)	Iran	Organizations	General	Daily decisions for long-term growth and survival	Short-term financial health, operational influence	3/5
36	Bagheri et al. (1)	Iran	Sport Bodies	National	Adaptive, globally oriented strategies	Complexity management, environmental scanning	3/5
37	Saber et al. (47)	Iran	Sport Organizations	National	Innovative strategy development	Execution of strategies	3/5
38	Kohandel et al. (6)	Iran	Sport Federations	National	Strategic orientation, human resource development, ethical strategy	Control mechanisms, learning strategy	4/5

MMAT Score: Mixed Methods Appraisal Tool (version 2018); maximum score: 5. Scores reflect quality appraisal (higher: more rigorous). No studies were excluded based on quality; scores inform weighting of conclusions. Strategic leadership focus: aspects related to vision, direction-setting, alignment, distributed leadership, organizational-level strategic functions. Management focus: operational, administrative, procedural, resource allocation aspects. Organization type categorized as: Federation (National Governing Body), Professional Club, League, National Sport Organization (NSO), International Federation, Olympic Program, Sport for Development, Elite Sport Environment. Level of competition based on the study’s stated context: Olympic (podium-level international), International (cross-national but not necessarily Olympic), National (domestic elite), Professional (commercial leagues), Nonprofit (community/SFD).

### 3.3 Prisma 2020 compliance

The PRISMA 2020-compliant flowchart (Figure 1) shows the exact numbers at each stage: records identified (*n* = 309), duplicates removed (*n* = 30), title/abstract records screened (*n* = 279), records excluded at title/abstract screening (*n* = 162), full-text reports sought (*n* = 117), full-text reports unavailable (*n* = 8), full-text reports assessed (*n* = 109), reports excluded at full-text assessment, with reasons (*n* = 71), and studies included (*n* = 38). Cohen's kappa for both screening stages was *κ* = 0.91 for title/abstract screening and *κ* = 1.00 for full-text screening, with explicit reporting of disagreements.

## 4 Discussion

The purpose of the current systematic review is to analyze, in an integrated manner, strategic leadership and performance management in sport organizations. The findings highlight a set of interlocking mechanisms ranging from leadership practices and strategic planning to performance management, sustainability, governance structures, and digital transformation systems. Therefore, for a better understanding, this discussion section is organized around three main levels emerging from the analysis: (1) governance and organizational structures, (2) leadership and human capital development, and (3) strategic, performance, and innovation systems.

The three thematic pillars emerged from a hybrid inductive-deductive analytic process. Deductively, we drew on configurational theory (5) and De Bosscher et al.'s (3) multi-level framework to anticipate that strategic leadership would manifest across structural, human, and processual dimensions. Inductively, we conducted thematic analysis following Braun and Clarke (12), with two reviewers independently coding all 38 studies. The three themes—Governance and Structure, Leadership and Human Capital, and Strategic and Performance Systems—reached saturation after 32 studies and were validated through reviewer triangulation (*κ* = 0.86 for theme assignment).

### 4.1 Sport governance and organizational structures

The following findings on governance and organizational structures are interpreted through the lens of strategic leadership. Governance mechanisms constitute the structural embodiment of strategic leadership at the organizational level, shaping how decisions are made, accountability is assigned, and resources are allocated (7).

#### 4.1.1 Key findings

Studies focusing on governance confirmed that sport organizations operate within complex systems in which public, private, and associative logics coexist. Several contributions show that governance performance rests on the combination of good governance principles (transparency, control, participation) and structural arrangements tailored to institutional contexts and stakeholder configurations (7). Empirical investigations in national and international federations reveal only partial implementation of good governance principles: democratization of processes and stakeholder identification are relatively advanced, whereas transparency, particularly the publication of annual reports, financial information, and strategic plans, remains underdeveloped (13). At the level of governance architectures, multiple configurations coexist. Analyses of presidential models show that “strong,” “managerial,” and “couple-based” presidencies can all be positively associated with sports and financial performance, suggesting contingency effects rather than a single optimal model (14). Meanwhile, other studies underscore the rise of collaborative approaches in federal networks, where board composition, voting systems, and leadership style condition the capacity to establish collaborative governance regimes between national and regional levels (O'Boyle et al. 53). At the international level, comparative QCA-based analyses indicate that there are multiple “paths” to high commercialization, combining staff specialization, social media engagement, and, in some configurations, lower levels of accountability (15). In addition, several studies highlight the structuring role of state frameworks and public policy. Documentary analyses of Lithuanian sport policy describe a bureaucratic, hierarchical configuration that is highly centralized around the ministry, which constrains innovation and generates tensions between procedural control and results orientation (13). By contrast, research on Ethiopian Olympic federations shows that the quality of strategic management and the fit between strategy, structure, and good governance practices are positively associated with perceived levels of good governance (16).

#### 4.1.2 Strategic leadership at the organizational level

These governance findings constitute strategic leadership at the organizational level in three ways. First, board composition and voting systems determine the distribution of strategic decision authority—a core strategic leadership function. Second, transparency mechanisms, or their absence, shape how strategic direction is communicated and legitimized across stakeholders. Third, the alignment between governance structures and institutional context represents strategic leadership's contingency principle—the ability to match organizational form to environmental demands (cf. contingency theory). Taken together, these findings support the view that sport governance should be conceptualized as a multilevel system linking macro-institutional constraints, meso-level structural choices, and micropolitical practices within boards and executive teams (17). These results are in accordance with the previous conclusions of De Bosscher's work. The body of work converges with institutional theory, stakeholder theory, and resource dependence perspectives by demonstrating how regulatory pressures, demands from public funders, sponsors, and media, and the quest for legitimacy shape governance architectures and strategic priorities (15). The coexistence of several presidential models, all associated with performance, calls into question the search for a single “best practice” template for federation governance (14). Instead, the evidence suggests that governance effectiveness depends on the alignment between the chosen model, available resources, the degree of professionalization of staff, and institutional culture, echoing contingency approaches in management (17). Similarly, analyses of collaborative regimes in federated structures show that reforms to board composition, the introduction of independent directors, and inclusive strategic planning processes can foster shared understanding and positive interdependence between governance levels (7). On the other side, studies on national sport policy also illuminate tensions between hierarchical bureaucratic logics and aspirations towards decentralization and NGO participation (13). This tension exemplifies a structural dilemma faced by many sport systems: how to reconcile the need to safeguard substantial public funds with the organizational agility required to respond to participant and community needs.

#### 4.1.3 Specific strengths and limitations

From a methodological standpoint, this level brings together studies that mobilize a wide range of designs: action research case studies, documentary analysis, survey-based inquiries, QCA, and structural mediation models (7, 15, 18). This diversity is a strength insofar as it allows triangulation of governance phenomena from complementary perspectives. Nonetheless, most studies are restricted to single nations or small case sets, which limits generalizability. The frequent reliance on self-reported data from senior officials without systematic triangulation with external stakeholders also introduces potential social desirability bias (13, 14). Finally, the predominantly cross-sectional nature of the evidence makes it difficult to capture temporal dynamics of reform and institutional learning. Furthermore, the geographical scope of the evidence base is limited. The majority of studies were conducted in European, North American, and Australian contexts, with fewer contributions from Africa, Asia, and South America. This constrains the transferability of findings and highlights a critical gap. Future research must investigate how strategic leadership is enacted and moderated within diverse cultural, economic, and political sport ecosystems.

#### 4.1.4 Technical factors in strategic leadership

While the previous sections focus on governance and structural conditions, technical factors (i.e., coach development, athlete pathways, talent identification, and scientific support) also constitute essential organizational conditions for strategic leadership. Several included studies directly address these technical dimensions. Mthombeni et al. (19) identified that coach provision, talent identification systems, and access to sport science support are critical enablers of success among elite athletes from historically disadvantaged areas. Hall et al. (20) demonstrated that the Talent Development Environment Questionnaire (TDEQ) can be used as a strategic tool to diagnose weaknesses in athlete development environments and guide targeted interventions. Truyens et al. (21) showed that configurational resource capability analysis, including talent identification, coach provision, competition access, and scientific support, is necessary to achieve international athletic success. These technical factors are not merely operational; they represent strategic leadership decisions at the organizational level regarding where to invest resources, how to structure development pathways, and which capabilities to prioritize.

### 4.2 Sport leadership and human capital development

#### 4.2.1 Key findings

Studies centered on leadership converge on the idea that sport organizations' performance is tightly linked to leadership styles and leadership development arrangements. In elite Olympic contexts, performance directors and national performance directors report leadership practices structured around four domains: vision, operations, people, and culture (22). These leaders stress the need to combine transformational behaviors, rigorous functional management, and strong relational skills to align actors around shared goals. In mass participation and university sport, several studies report that transformational leadership is perceived as dominant and positively correlated with organizational effectiveness, whereas laissez-faire leadership is negatively associated with effectiveness (23, 24). Other work shows that servant leadership fosters shared leadership, confirming the importance of empowering styles in sport for development organizations (25). Organizational culture emerges as a crucial vector for translating leadership orientations into practice. In Montenegrin clubs, a “team management” style combining task and people orientation is associated with a dominant clan culture characterized by trust, loyalty, and member development (26). Studies on organizational change also indicate that employees perceive hybrid styles combining flexibility, understanding, and, when necessary, authority as most effective, thereby reinforcing the pertinence of situational and contingency models (Şahin 60). Several contributions focus on leadership development. They underscore the centrality of experiential learning through assignments, cross-functional projects, and “crucible” experiences (27), the role of international development programs in the career trajectories of women leaders (28), and the construction of federation-specific strategic leadership models that integrate strategic orientation, control, human resource development, and ethical strategy (6, 29).

#### 4.2.2 Interpretation and theoretical positioning

Overall, the findings reaffirm the centrality of transformational, servant, and strategic approaches in contemporary sport environments. These approaches appear particularly suited to organizations characterized by high complexity, multiple stakeholder demands, and the need to continuously align resources with high-performance expectations (23, 25, 30). Yet the empirical evidence also shows that such styles are effective only in interaction with organizational culture, structure, and performance management systems, which is consistent with integrative perspectives linking leadership, climate, and management systems (26, 31). The importance attributed to experiential learning and the progressive construction of leadership capabilities reflects a shift from one-off training courses towards integrated talent development systems structured around individual development plans, project-based opportunities, and regular feedback (6, 27). These results resonate with human and social capital models in which leadership is conceived less as a fixed set of traits and more as a distributed capability built over time through experience and networks. However, some studies temper the explanatory weight of leadership by showing, for example, that organizational structure (number of employees, role held) is a stronger predictor of perceived organizational success than leadership styles or self-rated organizational effectiveness (32). This observation suggests that, in some contexts, human resource availability and role clarity may matter more than declared leadership styles, or that such styles are poorly captured by the measurement instruments used.

#### 4.2.3 Specific strengths and limitations

Leadership studies display several strengths: use of validated instruments (e.g., MLQ, SL7, SPLIT, TDEQ), application of multivariate analyses (multiple regression, SEM), and combination of qualitative and quantitative approaches that illuminate both practices and perceptions (20, 23, 25). Nonetheless, limitations are notable. Most designs are cross-sectional and rely on self-report data from leaders and followers, which constrains causal inference and raises concerns about common method bias (23, 32). Sample representativeness is often limited (small numbers of federations or clubs, overrepresentation of male leaders), restricting the transferability of findings to other cultural contexts and more diverse populations (25, 28). In addition, some studies exhibit serious psychometric weaknesses (low reliability for certain scales), which undermines the robustness of their conclusions (32).

### 4.3 Sport strategic management, performance and innovations

#### 4.3.1 Key findings

The third level shows that sport organizations are increasingly structuring their activity around sophisticated strategic management and performance systems that integrate resources, capabilities, and, more recently, sustainability and digital transformation concerns. Several studies report positive associations between strategic management quality (planning, analysis, implementation, control) and organizational performance in national federations and professional clubs (18, 24, 33). SEM-based models indicate that strategic planning explains a substantial share of the variance in perceived performance and that organizational structure partially mediates the relationship between strategic management and good governance (18, 24). From a performance management perspective, multiple contributions draw on frameworks such as SPLISS, ORFOC, multilevel performance management, and sport-adapted Balanced Scorecards (17, 21, 34). These studies converge on the idea that financial and material resources are necessary but not sufficient: configurations of resources and capabilities, including talent identification, coach provision, competition access, scientific support, and audiovisual strategies, condition international results and social diffusion of sport (19, 21, 34). Research on Olympic performance management further argues that performance is best understood as a dynamic system of interrelated processes linking strategic, operational, and individual levels (35). Methodological innovation is also visible in the development of diagnostic tools and integrated frameworks. The Talent Development Environment Questionnaire demonstrates that an ecological environment measure can identify specific weaknesses, guide targeted interventions, and document significant improvements over a 12-month period (20). Other work proposes holistic performance management models for national sport organizations that integrate macro, meso, and micro levels and underline the importance of feedback and feedforward loops (17). TQM/TQHRM approaches show, in tennis clubs, that the quality of management and human resources is strongly associated with overall performance via total quality human resource management (31). Two emerging areas stand out: sustainability and digital transformation. Delphi-based exercises on sustainable development priorities reveal expert consensus on the need to strategically prioritize sustainability, move beyond one-off actions, and embed sustainability requirements into bidding processes for sport events (36). Case studies on environmental sustainability integration show that structures of signification, legitimation, and domination, values, institutional logics, and resource allocation can simultaneously enable and constrain environmental embedding (37). In parallel, digital transformation is conceptualized as a lever for strengthening knowledge management (“digital knowledge management”), improving knowledge creation, storage, transfer, and application, and generating new forms of value (fan engagement, performance analytics, marketing segmentation) (38).

#### 4.3.2 Interpretation and theoretical positioning

From a resource- and capability-based perspective, these findings confirm that high-performing sport organizations are those able to structure resources into configurations that are coherent with their environment and objectives rather than merely accumulating assets (17, 21). Configuration analyses clearly demonstrate equifinality: multiple combinations of resources and capabilities can yield comparable performance levels, encouraging decision-makers to prioritize strategic coherence over simple imitation of “best practices” (15, 21). Equifinality, drawn from configurational theory (39, 40), refers to the principle that multiple distinct organizational configurations can achieve equivalent performance outcomes. In our model, equifinality is operationalized as follows: different combinations of governance type (e.g., presidential vs. managerial), leadership style (transformational vs. servant vs. distributed), and performance system design (e.g., SPLISS vs. Balanced Scorecard) can yield similar levels of organizational success, provided internal coherence and environmental fit are maintained. This principle is empirically supported by Clausen et al. (15) and Truyens et al. (21), both of which identified multiple “paths” to high performance. Performance management studies also emphasize that performance cannot be reduced to sporting results alone. The models reviewed integrate financial, organizational and social dimensions, as well as indicators of media exposure and participation, aligning with broader conceptions of the value created by sport organizations (17, 34). The use of ecological measures such as the TDEQ illustrates how environmental assessment can be more tightly coupled with intervention design and individual or team performance outcomes (20). Sustainability and digital transformation further expand this framework. The sustainability priority consensus indicates that international sport organizations recognize the need to move beyond symbolic initiatives towards long-term strategies embedded in decision-making processes and stakeholder relations (36). Studies on digital transformation suggest that technologies generate value only when accompanied by a supportive organizational culture, adequate skills, and robust data governance structures, reinforcing the knowledge-based view of knowledge as a strategic, hard-to-imitate resource (38).

#### 4.3.3 Specific strengths and limitations

This block exhibits substantial methodological density, with many studies employing correlations, multiple regression, structural equation modeling, and sophisticated comparative approaches (16, 24, 31, 33). Such rigor strengthens the evidence linking strategic planning, performance management, and organizational outcomes. Nonetheless, limitations persist: heavy reliance on perceived performance indicators, heterogeneity of composite indices, limited comparability across national contexts, and frequent absence of comparison groups or longitudinal designs restrict the strength of causal claims (34, 35, 38). The sustainability and digitalization fields remain predominantly explored through qualitative, exploratory work, which is appropriate given their emergent status but calls for more systematic quantitative and mixed-methods studies to validate proposed frameworks (36–38). Overall, even though strategic leadership has large benefits for sport organizations, the selected studies tend to focus only on a single leadership style and consequently fail to consider cross-cutting or underlying characteristics that might be present. Deeper future analyses of the traits of strategic leadership may enlighten the understanding of the reality of its practice within sport organizations.

#### 4.3.4 Toward a contingency model

**Figure 2:**
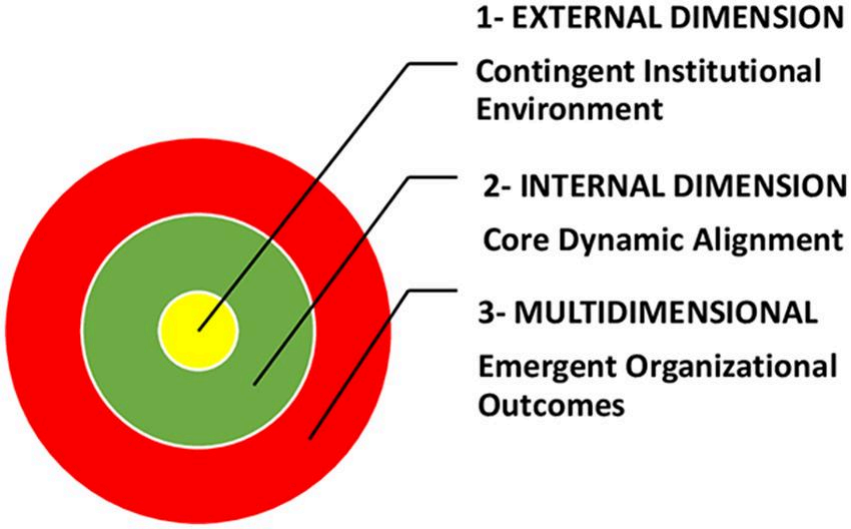
Contingency model of strategic alignment in elite sport organizations.

**Figure 3:**
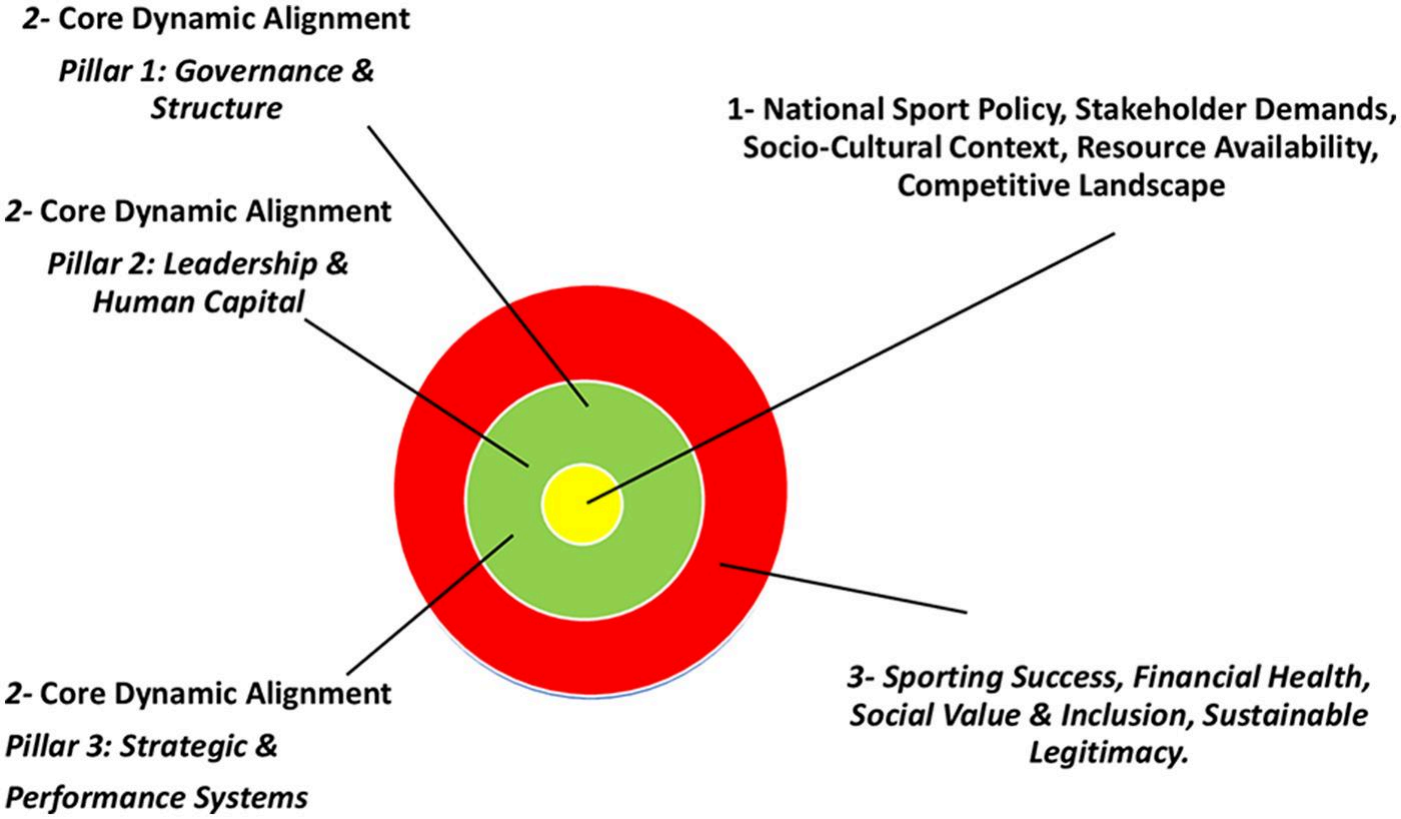
Dimensions of the contingency model of strategic alignment in elite sport organizations.

The thematic analysis reveals that governance structures, leadership development systems, and strategic performance mechanisms are not discrete factors but deeply interdependent. To move beyond a siloed understanding and to synthesize our findings, we propose a contingency model of strategic alignment in elite sport organizations (see Figures 2, 3). This model does not claim to represent strategic leadership as a singular phenomenon. Rather, it depicts how three organizational pillars, governance structures, leadership development systems, and strategic performance mechanisms, must achieve dynamic alignment for strategic leadership to emerge as an organizational property. Strategic leadership is the emergent outcome of this alignment, not any single pillar. The interrelationship of core pillars is detailed in Figure 3.

The model illustrates that the institutional context—encompassing national policy, stakeholder demands, and resource landscapes—establishes the boundaries within which sport organizations operate. Within these constraints, effective organizations achieve dynamic strategic alignment. This is not a static state but a continuous process of adjustment. For instance, a governance reform (e.g., introducing independent directors) must be matched by supportive leadership behaviors and integrated into strategic planning systems to be effective. Conversely, a leadership development initiative will falter without governance structures that empower leaders and performance systems that reward desired behaviors. The principle of equifinality, supported by our review, is central to the model. It suggests that there is no single “golden triangle” of governance, leadership, and strategy. Multiple configurations can lead to success, provided there is internal coherence among the pillars and fit with the external environment. The feedback loop from outcomes back to the core alignment represents organizational learning, allowing successful configurations to be reinforced and misalignments to be corrected. This model provides a holistic framework to guide future research and practice beyond the examination of isolated variables.

## 5 Conclusion

This review makes three distinct contributions. First, it shifts the unit of analysis from individual leaders to organizational systems, addressing a gap identified by Parent et al. (50). Second, it synthesizes three previously siloed literatures (governance, leadership, and strategy) into an integrated framework. Third, it proposes a testable contingency model with specific propositions for future research. By analyzing 38 studies, we have identified three interdependent pillars: governance and structure, leadership and human capital, and strategic and performance systems, which collectively underpin organizational success. The principal contribution of this review is the synthesis of these findings into the contingency model of strategic alignment in elite sport organizations (Table 4).

**Table 4:** Key factors in the contingency model of strategic alignment in elite sport organizations.

External dimension (institutional and contingent environment)
Key factors National Sport Policy, Stakeholder Demands, Socio-Cultural Context, Resource Availability, Competitive Landscape

Internal dimension (core dynamic alignment)
Pillar 1: Governance and structure Key factors Presidential models, board composition, accountability mechanisms, collaborative networks Pillar 2: Leadership and human capital Key factors Transformational/servant styles, experiential development, organizational culture, distributed capability Pillar 3: Strategic and performance systems Key factors Resource configuration (SPLISS/ORFOC), digital and sustainable innovation, performance management (TDEQ, BSC)

Multidimensional (emergent organizational outcomes)
Key factors Sporting success, financial health, social value and inclusion, sustainable legitimacy

The model makes explicit the central thesis of our review: high performance is not the product of any single leadership style, governance template, or strategic tool. It is an emergent property of dynamic organizational alignment. The literature decisively supports a contingency perspective, in which effectiveness is derived from the fit between internal configurations and the external institutional environment. In other words, based on the theoretical synthesis and strategic imperatives, the analysis yielded three critical theoretical and practical imperatives: (1) contingency over uniformity, (2) distributed leadership, and (3) configurational performance systems. First, contingency over uniformity in governance: the literature decisively favors contingency theory over a single institutional best practice. Governance effectiveness is not secured through the adoption of standardized principles alone, but through the strategic fit between structural arrangements (e.g., presidential models, accountability mechanisms) and the organization's unique institutional environment. A core tension remains the need to reconcile bureaucratic control (for transparency) with the organizational agility necessary for modern competition and innovation. Second, distributed leadership and cultural mediation: the efficacy of transformational and servant leadership styles is strongly supported, but their impact is shown to be highly contingent upon a mediating organizational culture and coherent structural supports. In this context, the concept of distributed leadership (41, 42) conceives leadership as an emergent property of social interactions and organizational systems, rather than a set of individual traits or behaviors. In elite sport organizations, this manifests through shared decision-making across boards, executives, coaches, and athletes. This pattern is supported by Svensson et al. (25) and Kohandel et al. (6). Leadership is best conceptualized as a distributed capability, built over time through integrated talent development systems that emphasize experiential learning and the progressive accumulation of human and social capital. Third, within configurational performance systems, high performance is fundamentally a configurational outcome, affirming the resource-based view and the principle of equifinality. Equifinality, as defined previously, is drawn from configurational theory (39, 40) and refers to the principle that multiple distinct organizational configurations can achieve equivalent performance outcomes. Performance systems must be designed to integrate a multidimensional range of indicators extending beyond athletic results to include financial health, social value, and critical emerging domains like sustainability and digital transformation. Value creation from these innovations is dependent on a supportive knowledge management culture and robust data governance. While this review offers a comprehensive map of the field, the reliance on predominantly cross sectional, self-reported data remains a methodological limitation, restricting the strength of causal claims and the capture of institutional evolution.

Therefore, further research must move toward several research directions. Research specifically should address four priorities. First, process inquiry: how does strategic alignment between governance, leadership, and performance systems evolve over time? Longitudinal panel studies tracking federations through reform periods (e.g., governance code changes) are needed. Second, organizational type comparison: do strategic leadership configurations differ systematically between federations, professional clubs, and leagues? A comparative configurational analysis could identify equifinal pathways specific to each organizational form. Third, measurement development: how can the “strategic leadership role of organizations” (e.g., policymaking, resource coordination, regulatory functions) be reliably measured? Fourth, cross-cultural validation: are the three-pillar model and equifinality principle invariant across diverse cultural, economic, and political sport systems? Replication studies in Asian, African, and South American contexts are urgently needed given the Western-centric evidence base.

Ultimately, the future success and legitimacy of sport organizations will depend on their capacity to achieve dynamic strategic alignment, effectively managing the complex, nonlinear relationships between robust governance, empowered human capital, and adaptive performance systems, as recommended in Table 5. Therefore, the agenda for leaders and researchers must shift from seeking best practices to managing strategic alignment. Future studies should use this model as a framework to examine the longitudinal processes of alignment and misalignment and to test how specific contextual factors moderate the relationships between its core components.

**Table 5:** Key recommendations for sport federations and leaders.

Model pillar	Theoretical imperative	Practical imperative	Supporting evidence
Governance and structure	Contingency over uniformity	Align governance models to institutional context	Ferkins et al. (7) and Ghodhbani et al. (14)
Leadership and human capital	Distributed leadership as emergent capability	Build leadership development systems, not just training	Frawley et al. (27) and Kohandel et al. (6)
Strategic and performance systems	Configurational performance design	Integrate financial, social, and athletic metrics	Kasale et al. (17) and Rodríguez et al. (34)
